# Have We Got Thy3 Wrong in the UK? A Four-Year Single-Site Analysis of Malignancy Rates in Thy3 Nodules

**DOI:** 10.7759/cureus.20125

**Published:** 2021-12-03

**Authors:** Bhargavi Chandrasekar, Sharan Jayaram, John de Carpentier

**Affiliations:** 1 Department of Otolaryngology, Lancashire Teaching Hospitals NHS Foundation Trust, Preston, GBR; 2 Department of Otolaryngology, Alder Hey Children's Hospital, Liverpool, GBR

**Keywords:** thyroid histology, thyroid cytology, diagnostic test accuracy, fine needle aspiration cytology (fnac), thyroid cancer, : thyroid nodule

## Abstract

Introduction

Thyroid nodules routinely undergo ultrasound-guided fine-needle aspiration (FNA), as recommended by the National Institute for Health and Care Excellence (NICE) and the British Thyroid Association (BTA). The cytology results are classified using the “Thy” system from Thy1 to Thy5. Intermediate Thy3 FNA results are challenging, as this suggests malignancy is possible, but the relatively low rates of malignancy can make decision-making difficult. Thy3 is further subdivided into Thy3a and Thy3f.

BTA recommends further ultrasound with or without FNA cytology for Thy3a nodules and hemithyroidectomy for Th3yf nodules based on a published positive predictive value (PPV) for malignancy of 17% for Thy3a and up to 40% for Thy3f results.

We aim to compare the actual malignancy rates of Thy3 nodules in our unit to these figures.

Methods

A retrospective study was performed looking at the histologically confirmed malignancy rates in Thy3a and Thy3f cytology over four years between January 2016 and December 2019.

Results

There were 162 separate Thy3 nodules in 156 patients included in this study, of which 60 were classified as Thy3a and 102 as Thy3f. 10% of patients with Thy3a nodules underwent repeat cytology. The histologically confirmed malignancy rate was 33% in Thy3a and 11% in Thy3f lesions.

Discussion

We found the rates of histologically confirmed malignancy are reversed compared to the published PPVs with a higher rate in Thy3a nodules and a lower rate in Thy3f. This suggests that the surgical decision-making and patient counselling may be based on flawed data in our unit and possibly throughout the UK, making a wider study involving multiple centers desirable.

## Introduction

Thyroid nodules, described by the American Thyroid Association, are “discrete lesions within the thyroid gland, radiologically distinct from surrounding thyroid parenchyma” [1]. The lifetime risk of developing a thyroid nodule is 5%-10% [2]. Thyroid nodules are important as they carry a risk of malignancy.

Thyroid cancer accounts for less than 1% of total cancer cases in the UK, but its incidence is rising. Since the early 1990s, the incidence of thyroid cancer has increased by 164%, with 3685 new cases of thyroid cancer diagnosed in the UK between 2015-2017. The incidence of thyroid cancer by age varies between males and females. The peak of incidence occurs in the 80 to 84 age group in men, and the peak in women is in the younger 40 to 49 age group [3].

Ultrasound-guided fine-needle aspiration (FNA) is recommended by the National Institute for Health and Care Excellence (NICE) and is a cost-effective investigation used in the management of thyroid nodules [2]. The Royal College of Pathologists (RCPath) provides guidance on the classification of FNA cytology using the Thy1-5 system [4]. This system was first described in 2004 and has been utilized in the UK since 2009.

An intermediate or Thy3 FNA result is challenging for the thyroid surgeon, as it suggests neoplasm is possible, but the nature of the lesion cannot be determined on cytology alone.

The British Thyroid Association (BTA) 2014 guidelines further subdivide Thy3 into two distinct categories, Thy3a and Thy3f. Thy3a suggests ‘there are atypical features present but not enough to place into any of the other categories. It cannot exclude follicular neoplasm or papillary carcinoma’. Thy3f is used when ‘a follicular neoplasm is suspected. The histological possibilities then include a hyperplastic nodule, follicular adenoma, or follicular carcinoma’ [4,5].

BTA recommends further ultrasound with or without FNA cytology for Thy3a nodules, with a multidisciplinary team (MDT) discussion if repeat sample is Thy3a, and directly proceeding to a diagnostic hemithyroidectomy for Th3yf.

The published positive predictive value (PPV) for malignancy by RCPath is 17% in Thy3a and up to 40% for Thy3f nodules, respectively [4,5].

This study aims to compare the malignancy rates of Thy3 nodules in our unit to national figures and guide our further management of Thy3 thyroid nodules.

This article was previously presented as a poster at the BAHNO 2021 Annual Scientific Virtual Meeting on May 14, 2021.

## Materials and methods

A retrospective, single-center, observational study was performed in our tertiary head and neck unit. Patients who had undergone an FNA between four years from January 2016 and December 2019 were identified from the hospital cytology database. Nodules that were graded Thy3a and Thy3f and patients with multiple nodules were included. Thyroid cytology graded Thy1-2, Thy4-5, or ‘Thy3’ only without subclassification into Thy3a or Thy3f were excluded. Children under the age of 18 were excluded as any subsequent surgical management was not performed at our center.

Histology results were available if the patient had subsequently undergone surgical excision of the corresponding thyroid lobe following the FNA. Information on further FNA sampling and histology outcomes were extracted from the hospital system 'Quadramed'. Due to the retrospective nature of the study, it was not possible to ensure that the histological category was related to the lesion which had undergone FNA if the surgically excised specimen had multiple nodules present. If histology data was available, it was categorized into one of the following: malignant (e.g., papillary carcinoma, medullary carcinoma), benign, or microcarcinoma.

Non-invasive follicular thyroid neoplasm with papillary-like nuclear features (NIFTP) was introduced in 2016 to replace the term ‘non-invasive encapsulated follicular variant of PTC’ and incorporated into WHO classification in 2017 [6,7]. Prior to this, all encapsulated follicular pattern tumours with nuclear features of papillary thyroid cancer (PTC) were classified as malignant irrespective of their invasive potential [8]. The new category aimed to reduce the overtreatment of such nodules by removing the term carcinoma [9]. However, these nodules are not truly benign, with the WHO classifying NIFTP as ‘unspecified, borderline or uncertain behaviour’ [7]. In our center, all histologically diagnosed cases of NIFTP were discussed at MDT and considered to be benign with little malignant potential. The consensus for management is monitoring as opposed to completion thyroidectomy and, therefore, NIFTP has been classified as benign in this study.

The positive predictive value (PPV) was calculated using all nodules that had a histological outcome available, so the denominator excluded nodules that were not surgically resected. The management of microcarcinomas is discussed on a case-by-case basis in our unit at MDT, and therefore two PPVs were calculated, one including and one excluding microcarcinomas. MDT outcomes and clinic discussions were obtained from the hospital system 'Evolve' and were reviewed.

Data was collected and analyzed using Microsoft Excel (Microsoft® Corp., Redmond, WA). No patient identifiable data was collected.

The audit was registered with and approved by the local audit department. Ethical approval was not deemed necessary as this was a retrospective audit of clinical outcomes, and research was not conducted on patients. 

## Results

There were 171 Thy3 nodules. Nine were classified as Thy3 only and not subclassified into Thy3a and Thy3f and therefore excluded. One hundred and sixty-two Thy3 nodules remaining in 156 patients were included in this study, of which 60 were classified as Thy3a and 102 as Thy3f. Six patients had bilateral Thy3 nodules. FNA cytology was reported by five individual pathologists. There was no “double reporting” whereby the Thy staging was independently reviewed by a second pathologist.

One hundred and thirty-one nodules were in females and 31 in males (F: M ratio 4.24:1). The average age was 52 years ranging from 19 to 86 years.

**Figure 1:**
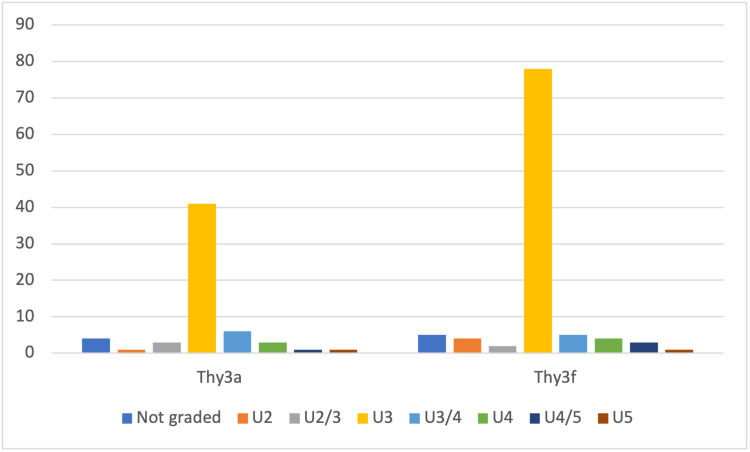
Ultrasound grading of the thyroid lesions of Thy3a and Thy3f nodules X-axis: ultrasound grading, Y-axis: number of ultrasounds

The majority of Thy3a and Thy3f nodules had been classified as U3 on ultrasound (68.3 and 76.5%) (Figure 1).

Thy3a

Of the 60 nodules, six (10%) underwent repeat FNA cytology. One also underwent an additional core biopsy (Table 1).

**Table 1:** Findings of repeat cytology in patients with Thy3a nodules

Repeat investigation	Repeat cytology result	Outcome
FNA	Thy1	Hemithyroidectomy – adenomatous nodule
FNA	Thy3f	Review in six months due to COVID-19
FNA	Thy3a	Watchful wait due to comorbidities
FNA	Thy3a	Hemithyroidectomy – papillary carcinoma, follicular variant
FNA	Thy3f	Surveillance – patient choice
FNA and Core	Thy4 and Anaplastic Ca/lymphoma	Total thyroidectomy – anaplastic/undifferentiated Ca

Forty-nine of the 60 (81.7%) patients underwent surgery, 46 hemithyroidectomies, and three total thyroidectomies. Excluding microcarcinomas, the malignancy conversion rate of Thy3a was 33%. Including microcarcinomas, it was 42%.

Thy3f

Surgery was performed in 95% of the Thy3f nodules (97 of 102). The malignancy conversion rate was 11%, excluding microcarcinomas, and 19%, including microcarcinomas.

**Figure 2:**
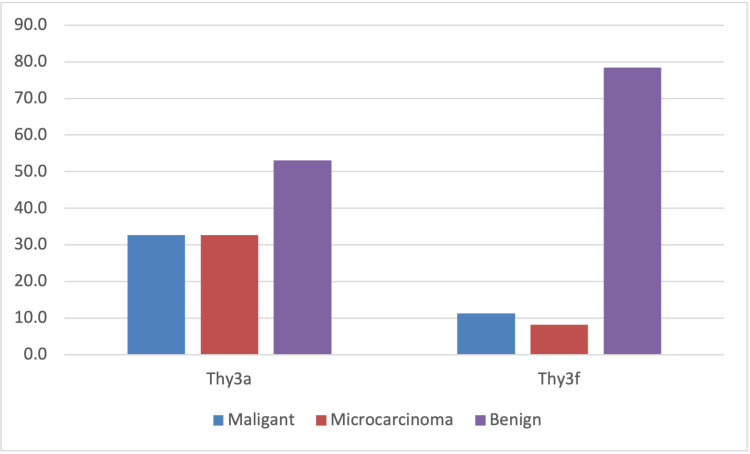
Histological outcomes following thyroidectomy X-axis: histological outcome, Y-axis: percentage of thyroid specimens

The malignancy conversion rate and histological outcomes are illustrated in Figure 2 and Table 2.

**Table 2:** Histological outcomes of surgically resected Thy3a and Thy3f nodules NIFTP (non-invasive follicular thyroid neoplasm with papillary-like nuclear features)

Cytology	Histology	Description	Number
Thy3a (n=49)	Malignant (n=16)	Papillary carcinoma	13
		Papillary carcinoma, follicular variant	2
		Anaplastic/undifferentiated carcinoma	1
	Microcarcinoma (n=2)	Papillary microcarcinoma	2
	Benign (n=31)	NIFTP	5
		Other	26
Thy3f (n=97)	Malignant (n=11)	Papillary carcinoma, follicular variant	4
		Papillary carcinoma	3
		Papillary oncoytic neoplasm of uncertain malignant potential	1
		Hurthle cell carcinoma	1
		Papillary carcinoma, cystic	1
		Medullary carcinoma	1
	Microcarcinoma (n=8)	Papillary microcarcinoma	8
	Benign (n=78)	NIFTP	5
		Other	76

## Discussion

Our study is a retrospective analysis in a single center of the rates of malignancy in patients undergoing thyroid surgery following Thy3a or Thy3f histology on FNA. We found that in our trust, the rates of histologically confirmed malignancy are reversed in comparison to the published PPV by RCPath. We found a substantially higher risk of malignancy for Thy3a nodules and a lower risk for Thy3f. This study, therefore, challenges the current advice given to patients and surgical decision-making in the UK.

The BTA introduced the subdivision of Thy3 into Thy3a and Thy3f in the 2014 guidelines [5]. Further assessment for Thy3a nodules, through repeat ultrasound-guided FNA sampling and MDT discussion, is currently recommended. In comparison, a diagnostic hemithyroidectomy is advised for Thy3f due to the higher quoted PPV. Despite BTA recommendations, only 10% of patients in this series with Thy3a cytology underwent further FNA sampling. This is presumably a result of the higher rates of malignancy observed informally by surgeons influencing clinical decision-making and possibly by patient preference. Only 16% of those who underwent further FNA sampling were downgraded. Therefore, it could be argued that repeat FNA sampling does not change the management plan in this group of patients. We acknowledge that this study represents a relatively small sample size. Other studies have demonstrated a much higher percentage of Thy3a nodules downgraded on repeat cytology [10,11]. One study reported 62% of repeat FNAs resulted in downgrading, but it had an equally small sample size [10].

Irrespective of the low rate of repeat FNA sampling, the PPV on Thy3a nodules in this study was 33% to 42%, presenting a strong case for diagnostic lobectomy being recommended as a first-line intervention. This PPV is much higher than the 17% published by RCPath and lies between rates reported by other similar studies (29% to 56.9%) [4,12-15].

Unlike Thy3a, we found the rate of malignancy in surgically managed Thy3f nodules to be lower than the up to 40% suggested in the RCPath guidance. In this sample, the rate of malignancy was 11% to 19%. This rate is also lower than others in the literature (29.2% to 64.8%) [12-15]. Thy3f is generally accepted to pose less of a decision-making challenge as its management has clear guidelines advising diagnostic lobectomy due to the presumed high PPV.

A recent systematic analysis of 13 papers (six from the UK), looking at histologically proven malignancy in nodules that had FNA cytology classified using the Thy system, reported a rate of malignancy of 25% in Thy3a nodules and 31% in Thy3f [16]. However, studies that only considered one Thy category or Thy3 subcategory (e.g., Thy3a and Thy3f only) were excluded, thereby limiting the sample size and overall reliability. Notably, some other published studies (which were excluded from the systematic review) have also observed higher rates of malignancy in Thy3a compared to Thy3f nodules in their series [12,13]. The findings in this four years, single-center sample combined with the lack of uniformity in the published evidence calls into question the UK guidelines.

Given these reversed findings compared to RCPath guidelines, we believe this study should be extended to involve multiple centers making the sample size greater and statistical significance more reliable. If this larger study confirms the findings in this study, the UK and BTA guidelines should be revisited and amended to reflect national data, thereby enabling surgeons to provide patients with reliable figures to assist decision-making during the consent process.

Two figures for PPV have been given in this study, one including and one excluding microcarcinomas. Other similar studies have excluded microcarcinomas in the calculation of risk of malignancy as they were reported to be incidental and did not belong to the target lesion [12,13].

We are not confident that this always applied to our study. The microcarcinomas found ranged in size from 1mm to 8mm. All histology specimens with microcarcinomas contained another larger benign nodule making a direct comparison between cytology and histology difficult. All cases were discussed at MDT, and the outcome for most was ultrasound surveillance of the remaining lobe in hemithyroidectomy cases. There was one exception when a patient with an 8mm microcarcinoma elected to undergo a completion thyroidectomy. We have therefore provided two figures for PPV, one excluding and the other including microcarcinomas.

The relatively small sample size is a limitation of the study but reflects the short duration (four years) of the study as a single trust. PPV was calculated comparing cytology and histology from patients that underwent surgical resections. Therefore, cases that were managed non-operatively were not included allowing for potential bias with an over or underestimation of the risk of malignancy. The theoretical variation from the PPV figures reported in this paper is likely to be low as surgery was offered to (and accepted by) a large proportion of patients in our unit who were acceptable candidates for surgery. This is reflected in the figures, 81.7% and 95% of the patients with Thy3a and Th3f nodules, respectively, underwent surgical resection. Despite the limitation of this study, the findings can be used to advise surgeons locally and inform patients of the risk of malignancy in patients who are suitable surgical candidates.

The application of the cytological Thy3 categories in our center compared to that in other centers is unknown and, therefore, a limitation of this study. The RCPath has published guidance on the distribution of Thy categories, estimating that 5%-10% and 14%-16% of nodules in the population will be graded Thy3a and Thy3f, respectively [4]. The use of the Thy3a and Thy3f categories has been reported to vary across centers, with the use of Thy3a ranging from 1.2% to 20.8% and Thy3f from 6.2% to 12.7%, respectively [10,14].

Interobserver bias can be a confounding factor, and Kojcan et al. found that there is a poor agreement for Thy3a [17]. It is therefore unknown if the high PPV for Thy3a in our study is accurate or a potential overuse of the category where lesions may have been bettered assigned as Thy3f or Thy4. It would be interesting and indeed desirable to assess whether there would be a rate of the Thy3a and Thy3f staging being altered (if at all) if a second pathologist independently double-reported the sample. This may be difficult to achieve due to the time constraints that pathologists find themselves under.

The authors are anecdotally aware that the pathologists who report these specimens often discuss the cytological appearances of these specimens with their colleagues, but as the report is “signed out” under a single pathologist’s name, it is impossible in a retrospective study to assess how often this occurred.

## Conclusions

This study highlights that in this sample, the rates of histologically confirmed malignancy are reversed compared to the RCPath predicated rates of malignancy in Thy3 cytology. This raises concern that surgical decision-making and patient counselling might be based on flawed data in our unit and possibly in the wider UK setting. These results suggest that in our unit, we should be offering further ultrasound with or without FNA cytology for Thy3f nodules and a diagnostic hemithyroidectomy for Th3ya nodules (the opposite of the BTA recommendations).
